# Biopsy-proven vancomycin-induced acute kidney injury: a case report and literature review

**DOI:** 10.1186/s12882-018-0845-1

**Published:** 2018-03-27

**Authors:** Anri Sawada, Kunio Kawanishi, Shohei Morikawa, Toshihiro Nakano, Mio Kodama, Mitihiro Mitobe, Sekiko Taneda, Junki Koike, Mamiko Ohara, Yoji Nagashima, Kosaku Nitta, Takahiro Mochizuki

**Affiliations:** 1 Department of Surgical Pathology, Tokyo Women’s Medical University, 8-1 Kawadacho, Shinjuku, Tokyo, 162-8666 Japan; 2 Department of Medicine Kidney Center, Tokyo Women’s Medical University, Tokyo, Japan; 3 Department of Nephrology, Kameda Medical Center, Chiba, Japan; 4 Department of Pathology, Kawasaki Municipal Tama Hospital, Kawasaki, Kanagawa Japan

**Keywords:** Acute kidney injury, Acute interstitial nephritis, Acute tubular necrosis, High-flux haemodialysis, Vancomycin

## Abstract

**Background:**

Vancomycin is the first-line antibiotic for methicillin-resistant *Staphylococcus aureus* and coagulase-negative strains. The risk of vancomycin-induced acute kidney injury increases with plasma vancomycin levels. Vancomycin-induced acute kidney injury is histologically characterized by acute interstitial nephritis and/or acute tubular necrosis. However, only 12 biopsy-proven cases of vancomycin-induced acute kidney injury have been reported so far, as renal biopsy is rarely performed for such cases. Current recommendations for the prevention or treatment of vancomycin-induced acute kidney injury are drug monitoring of plasma vancomycin levels using trough level and drug withdrawal. Oral prednisone and high-flux haemodialysis have led to the successful recovery of renal function in some biopsy-proven cases.

**Case presentation:**

We present the case of a 41-year-old man with type 1 diabetes mellitus, who developed vancomycin-induced acute kidney injury during treatment for Fournier gangrene. His serum creatinine level increased to 1020.1 μmol/L from a baseline of 79.6 μmol/L, and his plasma trough level of vancomycin peaked at 80.48 μg/mL. Vancomycin discontinuation and frequent haemodialysis with high-flux membrane were immediately performed following diagnosis. Renal biopsy showed acute tubular necrosis and focal acute interstitial nephritis, mainly in the medullary rays (medullary ray injury). There was no sign of glomerulonephritis, but mild diabetic changes were detected. He was discharged without continuing haemodialysis (serum creatinine level, 145.0 μmol/L) 49 days after initial vancomycin administration.

**Conclusions:**

This case suggests that frequent haemodialysis and renal biopsy could be useful for the treatment and assessment of vancomycin-induced acute kidney injury, particularly in high-risk cases or patients with other renal disorders.

## Background

Intravenous vancomycin (VCM) is the antibiotic of first choice for methicillin-resistant *Staphylococcus aureus* and *S. epidermidis* infections [1]. The increasing use of higher VCM doses has led to a higher incidence of VCM-induced acute kidney injury (AKI) [2], particularly in patients with risk factors such as hospitalization in the Intensive Care Unit, obesity, and pre-existing chronic kidney disease, although most of the evidence was based on observational studies [3]. The mechanism behind VCM-induced AKI is still uncertain; however, animal models and a few biopsy-proven cases have shown that VCM could induce acute interstitial nephritis (AIN) and/or acute tubular necrosis (ATN) [4]. A common strategy for preventing and treating VCM-induced AKI is monitoring plasma VCM levels and subsequent VCM withdrawal as necessary [3]. Haemodialysis, particularly high-flux haemodialysis, may be useful in a more effective removal of VCM in some cases [5–7]. Alternatively, oral prednisone has been tested in the cases of AIN [6, 8–11]. Here we present a biopsy-proven case of VCM-induced AKI in a patient with Fournier gangrene and type 1 diabetes mellitus (DM) and review previous published cases.

## Case presentation

**Fig. 1:**
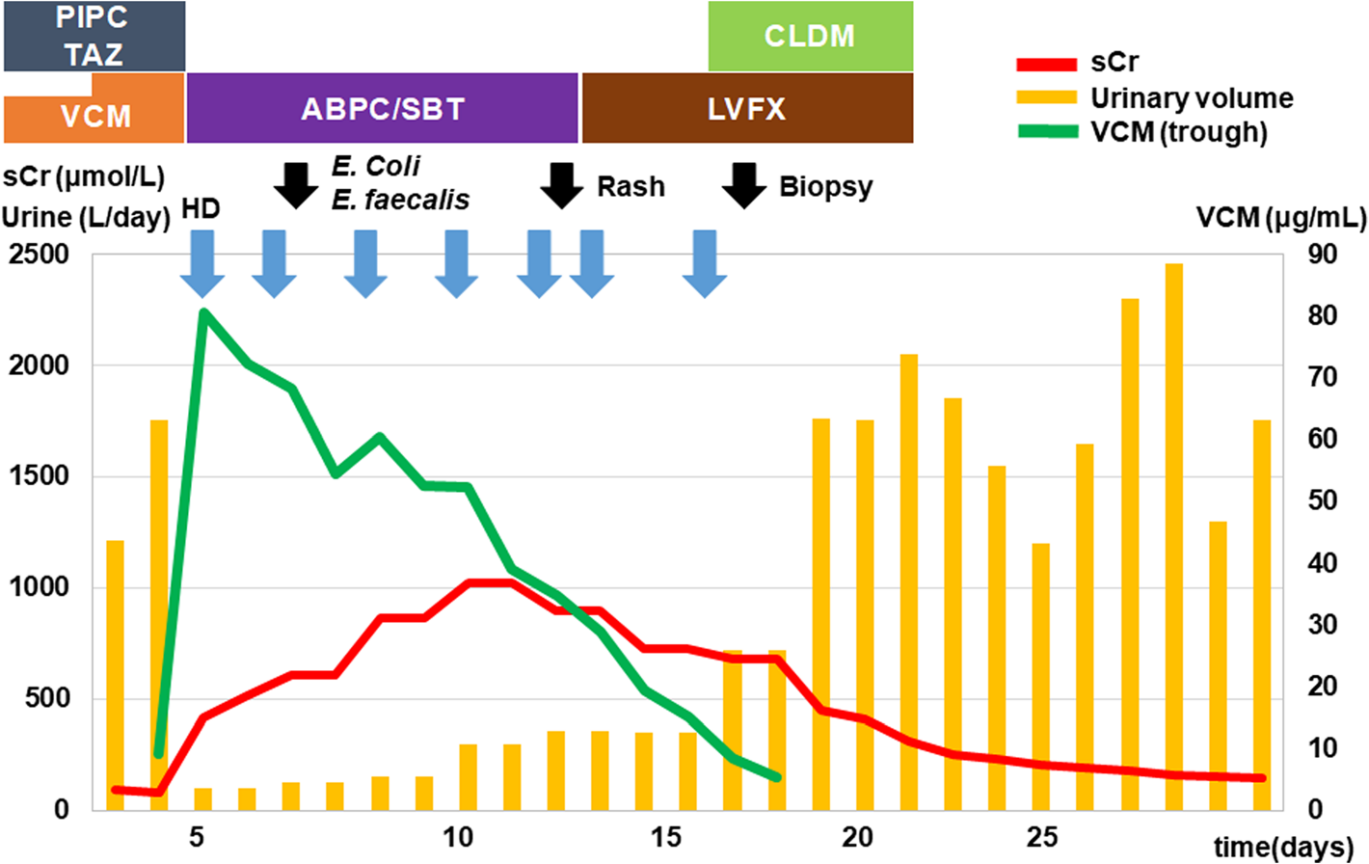
Clinical course for the treatment of Fournier gangrene and vancomycin-induced acute kidney injury in a 41-year-old man. The vertical biaxis shows the serum creatinine level (sCr, red), urinary volume (yellow), and plasma trough level of vancomycin (green). Intravenous vancomycin dosages were 3.0 g/day, then increased to 4.5 g/day. VCM, vancomycin; HD, haemodialysis, PIPC/TZA, Piperacillin/Tazobactam; ABPC/SBT, Ampicillin/Sulbactam; LVFX, Levofloxacin; CLDM, Clindamycin

**Fig. 2:**
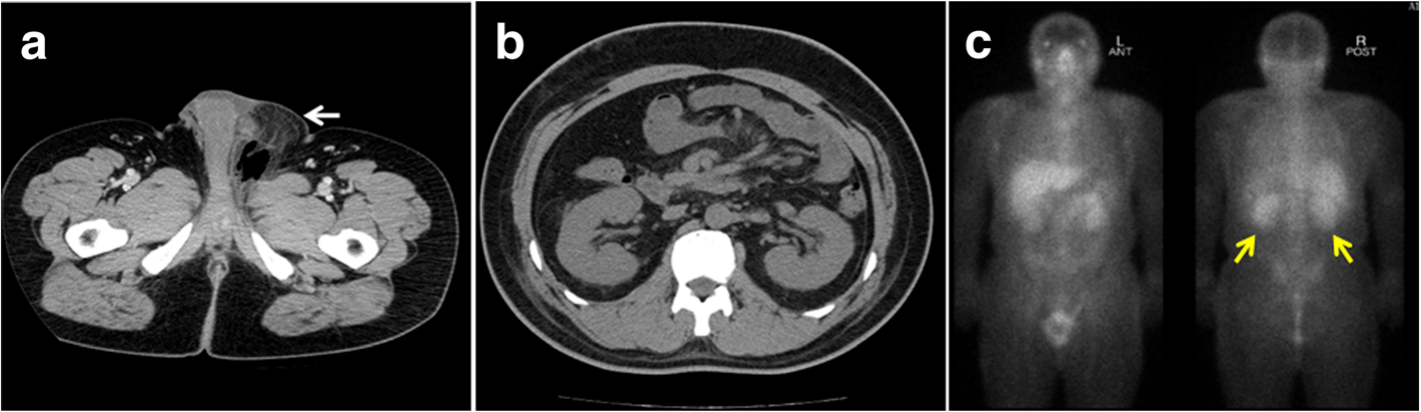
**a** Computed tomography image showing free air in the genital lesion (white arrow). **b** Computed tomography image showing no sign of hydronephrosis or renal atrophy. **c** Gallium scintigraphy showing significant accumulation in both kidneys (yellow arrows)

We present the case of a 41-year-old man who had been diagnosed with type 1 DM in junior high school. He was 168 cm tall and weighed 90.0 kg (body mass index, 31.9 kg/m^2^). His baseline serum creatinine (sCr) level was 79.6 μmol/L and his urinary protein level was 0.3 g/gCr. His blood pressure was well controlled with an aldosterone receptor blocker. DM control was poor (haemoglobin A1c 9.0–10.0%) under intensive conventional insulin therapy. His diabetic retinopathy was simple type. Pregabalin, duloxetine and mexiletine were also used for diabetic neuropathy. His family history was not significant except cerebral infarction in his grandmother. He initially visited a primary care unit because of general fatigue and high fever and was given oral levofloxacin. However, he later called an urgent care unit because of swelling and pain in his genitals. He was diagnosed with Fournier gangrene and admitted to our hospital (Fig. 1, clinical course). Table 1 showed urinary, blood and culture examination on admission. Inflammatory markers were elevated (white blood cell count 25,700/μL with left shift and C reactive protein 28.8 mg/L). Renal function was slightly abnormal (Blood urine nitrogen 22.0 mg/dL, sCr 91.1 μmol/L) and proteinuria was detected. Blood culture was negative. *Escherichia coli* and *Enterococcus faecalis* were detected from wound culture. Free air was noted in his genital area via computed tomography (CT) scan (Fig. 2a). He underwent debridement and received tazobactam/piperacillin (PIPC/TAZ) 4.5 g every 8 h and intravenous VCM 1.5 g every 12 h. Because his trough VCM level was still low (9.24 μg/mL, 15–20 μg/mL is for complicated infections [12]) and sCr stable (83.1 μmol/L) on day 3, intravenous VCM increased to 1.5 g every 8 h. Thereafter, he developed pitting pedal edema, weight gain (10 kg), reduced urine volume (100 mL/day), increased sCr (416.4 μmol/L) and trough VCM level (80.48 μg/mL) on day 6, which suggested VCM-induced AKI. Urinary examination results, which included N-acetyl-beta-D-glucosaminidase of 32.0 U/L, α1-microgloblin of 25.7 mg/L, and β2-microgloblin of 1800 μg/L, were also consistent with AKI. CT scan showed no signs of hydronephrosis or renal atrophy (Fig. 2b). Gallium scintigraphy showed significant accumulation in both kidneys (Fig. 2c).

**Table 1:** Laboratory data on admission

Complete blood cell count	
White blood cell (/μL)	25,700
Red blood cell (× 10^4^/μL)	476
Haemoglobin (g/dL)	13.6
Haematocrit (%)	38.6
Platelets (× 10^4^/μL)	20.7
Serum chemistries	
Total protein (g/dL)	6.1
Albumin (g/dL)	2.9
Blood urine nitrogen (mg/dL)	22.0
Creatinine (μmol/L)	91.1
Uric acid (mg/dL)	5.3
Sodium (mmol/L)	131
Potassium (mmol/L)	5.1
Chloride (mmol/L)	96
C reactive protein (mg/dL)	28.8
Haemoglobin A1c (%)	9.4
Urinalysis	
PH	5.5
Specific gravity	1.008
Protein	2+
Occult blood	–
Red blood cell sediment	1–4/hpf
White blood cell sediment	5–9/hpf
Cultivation	
Blood culture	negative
Wound culture	*E. coli, E. faecallis*

VCM and PIPC/TAZ were switched to ampicillin/sulbactam (ABPC/SBT), and frequent haemodialysis was performed on days 6–17, a total of seven times over 12 days (seven 4-h sessions with a blood flow rate of 120–150 mL/min and dialysate flow rate of 500 mL/min). Ethylene vinyl alcohol membrane was used on days 6 and 7, whereas polysulfone membrane was used on days 9, 11, 12, 14, and 17. His urine volume began to increase as his plasma VCM levels gradually decreased. A renal biopsy was performed on day 18 to rule out other renal disorders and evaluate for diabetic nephropathy. ABPC/SBT was switched to ciprofloxacin on day 13 because of a rash that developed mainly on his abdomen and back, and clindamycin was added on days 16–22. He was discharged on day 49 without haemodialysis and antibiotics (sCr, 145.0 μmol/L). Eight months later, his sCr was decreased to 109.6 μmol/L.

### Renal biopsy

**Fig. 3:**
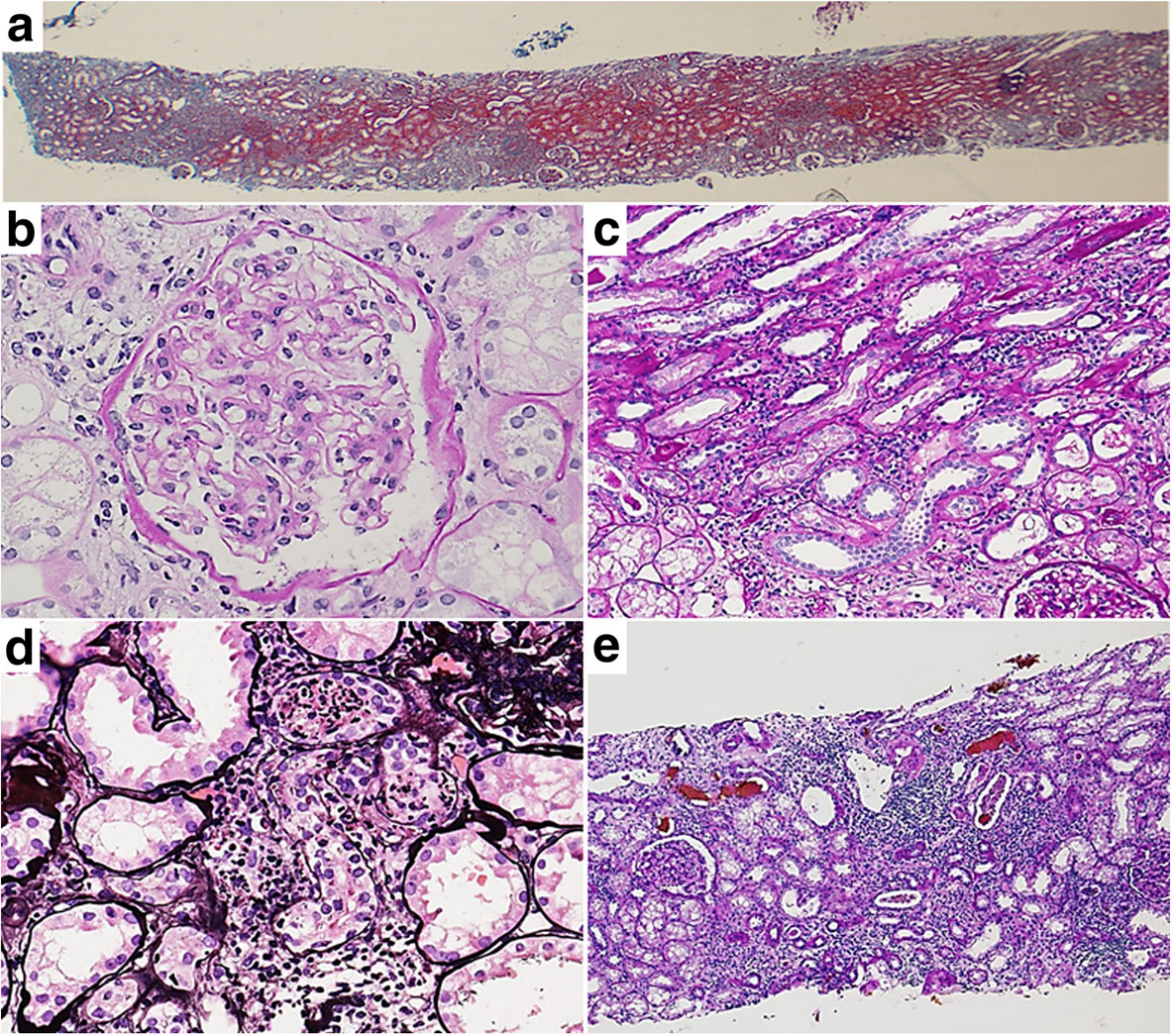
Kidney biopsy slide specimen showing: **a** subcapsular and medullary ray fibrosis in 10% of the specimen, **b** mild mesangial expansion in the glomeruli, **c** focal but severe lymphocyte infiltration and tubulitis, **d** tubular epithelium injury with nuclear denudation or tubular dilatation, and **e** interstitial monocyte infiltration and tubulitis mainly distributed in the medullary ray lesion [**a**, Masson trichrome, × 2; **b**, **c**, Periodic acid–Schiff, × 40 and × 20, respectively; **d**, Periodic acid–methenamine–silver, × 40; **e**, Tamm–Horsfall protein staining added on Periodic acid–Schiff, × 10]

The specimen included 16 glomeruli with cortex (no medulla). Subcapsular and medullary ray fibrosis was found in 10% of the specimen on Masson staining (Fig. 3a). Glomeruli showed no sclerotic or inflammatory changes, but mild mesangial expansion without significant depositions of immunoglobulin or complement in immunofluorescence was found. Nodular lesions were not detected (Fig. 3b). Focal but severe AIN (Fig. 3c) and tubular epithelium injury with nuclear denudation or tubular dilatation (ATN) (Fig. 3d) were detected. Interstitial monocyte infiltration and tubulitis were mainly distributed in the medullary ray lesions (Fig. 3e). There were no obvious eosinophilic infiltrations or granular lesions in the specimen. Mild intimal fibrosis was found in some of the small interlobular arteries, and mild hyalinosis was also noted in an arteriole. In summary, the kidney biopsy showed that ATN and focal AIN with mild diabetic nephropathy.

## Discussion

In general, an AKI episode is an independent risk factor for end-stage renal disease and death, and patients with pre-existing chronic kidney disease (CKD) are at higher risk for long-term mortality and dialysis after hospital discharge [13].

The first case series of VCM-induced AKI was reported in 1958 [14]. The incidence of VCM-induced nephrotoxicity was reported in approximately 5% of patients [2]. VCM-induced AKI is initially diagnosed when 50% sCr (or 44.2 μmol/l) elevation from baseline is detected in at least two different time points after administration of VCM treatment [15]. However, many of recent studies are committed to the definition and classification of AKI of RIFLE, AKIN and KDIGO criteria [16–18].

Plasma VCM level should be controlled in the appropriate range to prevent VCM-induced AKI. Plasma VCM level could be measured with therapeutic drug monitoring (TDM), such as VCM trough and area under the curve (AUC). However, VCM trough and AUC might be insufficient for prediction of VCM-induced AKI in large population study [19]. Moreover, available TDM guidelines still need optimizations to establish a more reliable VCM-TDM strategy in accordance with risk factors [20]. Previous studies showed that minimal sCr elevation is associated with prognosis of AKI [21], and intensive monitoring of urine output could be useful for AKI diagnosis and better outcomes [22]. More careful monitoring should be used to detect AKI as soon as possible in VCM usage.

Although VCM nephrotoxicity was well known in clinical settings, only 12 cases of biopsy-proven VCM-induced AKI have been reported so far as renal biopsy is rarely performed for such cases (Table 2) [4–11, 23–25]. Most of them showed AIN, and three cases showed ATN. Renal biopsy in our case (peak sCr, 1020.1 μmol/L) and one of the reported cases (peak sCr, 1034.3 μmol/L) revealed ATN and AIN. Diabetic nephropathy [11], IgA nephropathy [11], and lupus nephritis [25] were found simultaneously.

**Table 2:** Cases of biopsy-proven vancomycin induced AKI

Case	Patient Characteristics (age/sex/infections)	Complications	Baseline Cr (μmol/L)	Peak Cr (μmol/L)	Biopsy	Treatment (+ VCM withdrawal)	Final follow up Cr (μmol/L)
①^4^	79/F CNS bacteremia		79.6 – 97.2	1034.3	ATN+AIN	PSL	88.4
②^5^	*67/M* *S. aureus* endocarditis		132.6 –176.8	583.4	AIN	HD	death
③^6^	70/M MRSA abscess	TEN	106.1	848.6	AIN	HD+PSL	death
④^7^	8/M Infection of VP shunt		35.3	176.8	ATN	HD	35.4
⑤^8^	63/M Sternal wound dehiscence	CAD	53.0 – 132.6	839.8	AIN	HD+PSL	106.1
⑥^9^	44/M Osteomyelitis	DM	ND	751.4	AIN	HD+PSL	247.5
⑦^10^	51/M Osteomyelitis		79.6	503.9	AIN	PSL	114.9
⑧^11^	45/F Osteomyelitis	Type 2 DM	106.1	203.3	AIN DMN	PSL	168.0
⑨^11^	61/M Surgical infection	Spinal stenosis	88.4	627.6	AIN IgAN	PSL	212.2
⑩^23^	*35/M* *S. aureus* loculated pleural effusion		ND	574.6	AIN	ND	114.9
⑪^24^	71/F MRSA bacteremia		70.6	397.8	ATN	HD	HD
⑫^25^	13/M Toxic skin syndrome	SLE	106.1	495.0	ATN LN type V	PSL (for LN)	79.6
This case	41/M Fornier disease	Type1 DM	79.6	1020.1	ATN+AIN DMN	HD	109.6

*M* male, *F* female, *VP* ventriculoperitoneal, *MRSA* methicillin - resistant *Staphylococcus aureus, CNS* coagulase negative *Staphylococcus aureus, DM* diabetes, *SLE* systemic lupus erythematosus, *TEN* toxic epidermal necrolysis, *CAD* coronary artery disease, *ND* not described, *Cr* creatinine, *LN* lupus nephritis, *AIN* acute interstitial nephritis, *ATN* acute tubular necrosis, *HD* hemodialysis, *VCM* vancomycin, *PSL* prednisolone

Although the mechanism of kidney injury is still unknown, VCM induced oxidative stress that promotes reactive oxygen species was thought to be the main one [26]. Animal study showed that VCM induced renal tubular injuries were ameliorated by the use of antioxidants [26, 27]. An allergic reaction could be responsible to VCM induced AIN [11]. A case of recurrent AIN after secondary challenge of VCM also implied such an immunologic reaction [28].

Meanwhile, it is difficult to define what is cause of VCM-induced AKI in clinical cases; synergistic toxicity of VCM and other antibiotics such as PIPC/TAZ, cefepime, aminoglycoside should be considered in the current case as shown in previous studies [29–31]. Other multiple drug usages could affect VCM pharmacokinetics. Furthermore, VCM-induced AKI is more likely to occur in pre-existing renal disease [3].

In our case, nephrotoxicity of VCM could be enhanced in combination with PIPC/TAZ, which can decrease VCM clearance, and elevate plasma VCM level [29]. Renal biopsy was necessary in our case, because we need to differentiate other renal diseases such as glomerulonephritis and diabetic nephropathy for proteinuria on admission and continuous oliguria. The renal biopsy showed ATN and localized AIN with mild diabetic nephropathy, which suggested that the main cause of AKI was considered to be VCM induced ATN and AIN. AIN caused by ABPC/SBT should be considered, because this case showed rash. Although ABPC/SBT frequently cause rash (1.2%), severe AKI is rare in ABPC/SBT usage by itself [32, 33].

In previous cases of biopsy-proven VCM-induced AKI, diffuse or focal ATN was related to relatively higher levels of sCr [4, 7, 24, 25], and one case with severe ATN needed to continue haemodialysis for at least 2 months after treatments [24]. Oral prednisone therapy has been attempted in AIN cases [6, 8–11], but has not been established for VCM-induced AKI. Oral prednisone therapy was not attempted in our case because he had poor controlled DM and frontier gangrene. Renal biopsy result (localized AIN) was commit to our decision on no steroid use in the retrospective view. However, if AIN lesion is more expanded, oral prednisone should be considered to treat not only for VCM-induced AKI but for possible side effects of ABPC/SBT.

Haemodialysis, particularly high-flux haemodialysis, might be useful in a more effective removal of VCM in some cases [5–7]. In paediatric cases, reducing plasma VCM levels by high-flux haemodialysis contributed to the good renal prognosis [7, 34]. In our case, frequent haemodialysis with high-flux filters was performed seven times over 12 days, and urine volume was increased right after plasma vancomycin level was decreased (Fig. 1).

VCM is composed of a large glycopeptide compound (molecular weight, 1450 Da) [35] with a heterogeneous protein-binding rate (24.3–64%) [36]. Although conventional haemodialysis membranes such as cuprophan cannot adequately remove VCM (removal rate, 6%) [37], high-flux filters such as polysulphone, polynitrile, and polymethylmethacrylate can remove VCM from patients more effectively (35–46%) [37, 38]. However, rebounding of plasma VCM 3–6 h after haemodialysis was also reported [37], which suggests that frequent haemodialysis could be more useful. In our case, removal rates of plasma VCM were 10.3–20.3% using ethylene vinyl alcohol membrane and 13.2–35.2% using polysulfone membrane.

## Conclusion

In summary, we present an adult patient with type 1 DM who developed VCM-induced AKI during treatment for Fournier gangrene. Early diagnosis and treatment led to the successful recovery of his renal function. This case suggests that more careful VCM-TDM and intensive monitoring of sCr and urinary output to detect AKI should be considered in VCM usage. For the diagnosis, renal biopsy of VCM induced AKI is useful to assessment prognostic and therapeutic option in cases at a high risk or those with other renal disorders. For the treatment, frequent haemodialysis could be useful in high concentration plasma VCM.

## Abbreviations

ABPC/SBT: Ampicillin/sulbactam
AIN: Acute interstitial nephritis
AKI: Acute kidney injury
ATN: Acute tubular necrosis
CKD: Chronic kidney disease
CT: Computed tomography
DM: Diabetes mellitus
PIPC/TAZ: Tazobactam/piperacillin
sCr: Serum creatinine
VCM: Vancomycin
